# Abdomen anatomic characteristics on CT scans as predictive markers for short-term complications following radical resection of colorectal cancer

**DOI:** 10.3389/fsurg.2022.899179

**Published:** 2022-07-18

**Authors:** Xiao Zhang, Zhengyang Yang, Cong Meng, Jiale Gao, Yishan Liu, Bohao Shi, Liting Sun, Guocong Wu, Hongwei Yao, Zhongtao Zhang

**Affiliations:** Department of General Surgery, Beijing Friendship Hospital, Capital Medical University & National Clinical Research Center for Digestive Diseases, Beijing, China

**Keywords:** Colorectal cancer, postoperative complication, abdomen anatomic characteristic, propensity score match, complication prediction

## Abstract

**Background:**

Prediction and management of short-term postoperative complications in patients with colorectal cancer are essential in postoperative rehabilitation. Through CT scan images, we can easily measure some parameters of abdomen anatomic characteristics. This study aimed to assess whether there is a relationship between the abdomen anatomic characteristics and short-term postoperative complications.

**Materials and methods:**

We conducted a retrospective study. Eighty patients in each complication group and non-complication group were recruited with propensity score match. Demographics, perioperative laboratory results and surgical information were collected and compared between groups with univariate analysis. Significant elements were brought into subsequent logistic regression analysis and ROC analysis for further identification.

**Results:**

Univariate analysis showed that preoperative white blood cells, preoperative neutrophil counts, rectus abdominis thickness (RAT), subcutaneous fat thickness (SFT), and abdomen depth (AD) were significantly different between the complication group and non-complication group. Logistic regression analysis demonstrated that higher RAT (*p* = 0.002), SFT (*p* < 0.001) and AD (*p* < 0.001) independently predicted the incidence of short-term postoperative complications.

**Conclusions:**

In this study on patients undergoing radical resection of colorectal cancer, abdomen anatomic characteristics including higher RAT, SFT and AD are associated with an increased risk of short-term postoperative complications.

## Introduction

Colorectal cancer is one of the most common malignancies worldwide, accounting for approximately 10% of cancer cases and deaths (1, 2). Surgical resection is the principal measure to treat colorectal cancer. However, postoperative complication rate differs between 10% and 37%. Some studies demonstrate that postoperative complications are associated with long-term survival (3). Therefore, it is of great significance in clinical practice to discover the predictive marker of complications and identify the occurrence of complications early.

As a necessary imaging diagnosis method for colorectal cancer, CT scan also contains much information on abdomen anatomic characteristics, including rectus abdominis thickness (RAT), subcutaneous fat thickness (SFT), abdomen depth (AD), and abdomen width (AW). There are very few studies focusing on the effect of these factors on postoperative complications. Therefore, we conduct this retrospective study to investigate the associations between abdomen anatomic characteristics on CT scans and short-term postoperative complications.

## Materials and methods

Consecutive patients who received radical resection of colorectal cancer in our hospital from January 2018 to June 2021 were included in our analysis. We retrospectively collected their clinical data. The inclusion criteria were: (1) patients age ≥ 18 years old. (2) patients who received radical resection of colorectal cancer. (3) patients with a definitive pathological diagnosis of colorectal cancer. The exclusion criteria included: (1) patients without definite pathological diagnosis; (2) patients whose preoperative CT scan images are unavailable in medical record analysis or information management system of the radiology department; (3) patients without complete perioperative information or explicit information about short-term complications.

The clinical data, including demographics, perioperative laboratory results, and surgical information, was collected from the Electronic Medical Record System. Demographics include sex, age, hypertension, diabetes, other underlying diseases, abdominal surgical history, tobacco usage, alcohol usage, and body mass index (BMI). Preoperative laboratory results include albumin (ALB), alanine transaminase (ALT), aspartate transaminase (AST), triglyceride, total cholesterol (TC), white blood cell (WBC), neutrophil counts, lymphocyte counts, monocyte counts, haemoglobin (Hb), platelet (PLT), C-reactive protein (CRP), prothrombin time (PT), D-Dimer, alpha-fetoprotein (AFP), carcinoembryonic antigen (CEA), CA125, CA199, and CA724. All these results are from the final blood test results before surgery. Postoperative laboratory results, which are derived from the first blood test results after the surgery, including postoperative ALB and postoperative CRP. Surgical information includes operation time, intraoperative haemorrhage, intraoperative blood transfusion, and total hospitalization time.

**Figure 1:**
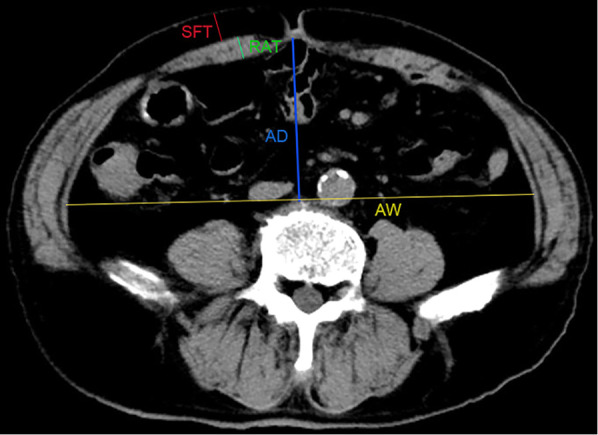
Measurement of abdominal anatomic characteristics on CT images. RAT: the maximum sagittal distance from the top to the visceral side of rectus abdominis; SFT: the maximum sagittal distance from the top to the visceral side of the subcutaneous fat; AD: the distance between the bottom of umbilicus and the top of vertebra; AW: the maximum transverse distance of the abdominal cavity perpendicular to the measurement line of the AD.

Abdomen anatomic characteristics, including rectus abdominis thickness (RAT), subcutaneous fat thickness (SFT), abdomen depth (AD), and abdomen width (AW), were measured by two doctors independently and averaged using the information management system of the radiology department. To standardize the measurement results, all the values were measured at the umbilicus level of the last preoperative supine CT images. RAT is the maximum sagittal distance from the top to the visceral side of the rectus abdominis. SFT is defined as the maximum sagittal distance from the top to the visceral side of the subcutaneous fat; AD is defined as the distance between the bottom of the umbilicus and the top of the vertebra; AW is defined as the maximum transverse distance of the abdominal cavity perpendicular to the measurement line of the AD (Figure 1).

Short-term complications are defined as any condition requiring conservative or surgical treatment occurring within 30 days after the operation. We got this information through a retrospective review of electronic medical record systems and postoperative follow-up records.

Statistical analysis was conducted using SPSS software (IBM SPSS Statistics, version 25.0). All the analysis was two-tailed. The confidence interval was 5–95%, and *p*-values < 0.05 was considered a significant statistical difference. Quantitative data was given as the median ± SD (standard deviation) and analysed using an unpaired t-test with Welch's correction. Categorical variables were presented as frequency (percentage) which were analysed using chi-square with Fisher's exact test. Variables with statistical differences between groups identified by univariate analysis were further assessed by logistic regression analysis. We performed receiver operating characteristic (ROC) analysis to evaluate prediction ability and an optimal cut-off value of the relative variables.

## Results

From January 2018 to June 2020, 216 eligible patients who received radical resections of colorectal cancer were included in our analysis. According to the occurrence of short-term postoperative complications, patients were divided into two groups, the complication group, and the non-complication group. Because of the high heterogeneity, we conducted propensity score matching to make them comparable. Each group contained 80 patients. The propensity score model included all demographic data: age, gender, hypertension, diabetes, other underlying diseases, past abdominal surgical history, tobacco usage, alcohol usage, and BMI. After propensity score matching, there were no significant differences in demographic information between the two groups, as shown in Table 1.

**Table 1:** Demographics of patients between complication group and non-complication group after propensity score matching.

	Complication group	Non-complication group	*p* value
Gender			
Male (*n*, %)	49 (61.3%)	44 (55%)	0.423
Age (mean ± SD)	63.59 ± 11.13	63.96 ± 11.40	0.834
Hypertension (*n*, %)	35 (43.8%)	33 (41.3%)	0.749
Diabetes (*n*, %)	16 (20%)	18 (22.5%)	0.699
Other underlying disease (*n*, %)	37 (46.3%)	37 (46.3%)	1
Past abdominal surgical history (*n*, %)	20 (25%)	22 (27.5%)	0.719
Tobacco usage (*n*, %)	24 (30%)	22 (27.5%)	0.727
Alcohol usage (*n*, %)	19 (23.8%)	17 (21.3%)	0.705
BMI (mean ± SD)	23.96 ± 3.76	23.60 ± 3.08	0.52

### Preoperative laboratory data

Compared to the non-complication group, the complication group showed higher white blood cells (6.43 ± 2.89 vs. 5.65 ± 1.44, *p* = 0.033) and higher neutrophil counts (4.23 ± 2.71 vs. 3.44 ± 1.21, *p* = 0.020), with the normal range of white blood cells 3.5–9.5 and neutrophil counts 1.8–6.3. No other statistical differences were found between the complication group and the non-complication group. (Table 2)

**Table 2:** Perioperative laboratory results between complication group and non-complication group after propensity score matching.

	Complication group	Non-complication group	*p* value
Preoperative ALB (mean ± SD) g/L	37.37 ± 4.23	37.81 ± 3.45	0.474
Preoperative ALT (mean ± SD) U/L	15.60 ± 13.74	14.93 ± 7.95	0.706
Preoperative AST (mean ± SD) U/L	18.10 ± 7.33	18.62 ± 5.32	0.61
Preoperative triglyceride (mean ± SD) mmol/L	1.47 ± 0.80	1.42 ± 0.61	0.621
Preoperative TC (mean ± SD) mmol/L	4.50 ± 0.99	4.50 ± 1.15	0.963
Preoperative WBC (mean ± SD) 10^9^/L	6.43 ± 2.89	5.65 ± 1.44	0.033*
Preoperative neutrophil counts (mean ± SD) 10^9^/L	4.23 ± 2.71	3.44 ± 1.21	0.02*
Preoperative lymphocyte counts (mean ± SD) 10^9^/L	1.77 ± 1.49	1.82 ± 1.82	0.837
Preoperative monocyte counts (mean ± SD) 10^9^/L	0.43 ± 0.33	0.40 ± 0.12	0.429
Preoperative Hb (mean ± SD) g/L	123.70 ± 22.57	123.75 ± 19.76	0.988
Preoperative PLT (mean ± SD) 10^9^/L	249.61 ± 92.21	231.84 ± 73.78	0.18
Preoperative CRP (mean ± SD) mg/L	11.05 ± 35.80	12.86 ± 22.67	0.834
Preoperative PT (mean ± SD) s	11.62 ± 0.73	11.46 ± 1.45	0.363
Preoperative D-Dimer (mean ± SD) ug/ml	0.74 ± 0.54	0.90 ± 0.93	0.184
Preoperative AFP (mean ± SD) ng/ml	3.03 ± 1.42	7.02 ± 39.01	0.368
Preoperative CEA (mean ± SD) ng/ml	13.88 ± 32..53	7.22 ± 11.93	0.093
Preoperative CA125 (mean ± SD) U/ml	14.65 ± 22.83	17.51 ± 46.53	0.627
Preoperative CA199 (mean ± SD) U/ml	47.94 ± 226.78	23.18 ± 53.41	0.349
Preoperative CA724 (mean ± SD) U/ml	4.74 ± 6.14	4.03 ± 4.55	0.434

### Clinical information relevant to the surgery

75% of patients in the complication group and 82.5% of patients in the non-complication group received laparoscopic surgery, without a statistical difference. As for other clinical information relevant to surgery, there are no statistical differences between the two groups in tumour location, stoma, operation time, intraoperative haemorrhage, intraoperative blood transfusion, postoperative CRP, and ALB. Obviously, the total hospitalization time of the complication group is significantly higher than that of the non-complication group (23.18 ± 10.15 vs. 15.65 ± 4.17, *p* < 0.001). (Table 3)

**Table 3:** Clinical information relevant to surgery between complication group and non-complication group after propensity score matching.

	Complication group	Non-complication group	*p* value
Surgical approach (*n*, %)			0.246
Laparoscopy	60(75%)	66(82.5%)	
Open	20(25%)	14(17.5%)	
Tumor location (*n*, %)			0.094
Ascending	18(22.5%)	25(31.2%)	
Transverse	8(10.0%)	2(2.5%)	
Descending	7(8.8%)	2(2.5%)	
Sigmoid	17(21.2%)	21(26.3%)	
Rectal	30(37.5%)	30(37.5%)	
Stoma (*n*, %)			0.385
Yes	26(32.5%)	21(26.3%)	
No	54(67.5%)	59(73.7%)	
Operation time (mean ± SD) min	184.14 ± 85.66	162.52 ± 88.63	0.12
Intraoperative haemorrhage (mean ± SD) ml	115.88 ± 183.63	106.69 ± 156.44	0.737
Intraoperative blood transfusion (*n*, %)	10 (12.5%)	4 (5%)	0.162
Postoperative CRP (mean ± SD)	44.08 ± 33.64	38.86 ± 30.35	0.399
Postoperative ALB (mean ± SD)	31.83 ± 4.22	32.49 ± 3.67	0.29
Total hospitalization time (mean ± SD) day	23.18 ± 10.15	15.65 ± 4.17	<0.001*

### Data on abdomen anatomic characteristics

In the course of our analysis, we chose to measure the following parameters to represent the abdomen anatomic characteristics: the SFT, the RAT, the AD, and the AW. The statistical results of these four parameters are shown in Table 4. We found that the complication group had higher SFT (2.72 ± 0.82 vs. 2.28 ± 0.89 *p* = 0.001), higher RAT (1.27 ± 0.28 vs. 1.01 ± 0.24, *p* < 0.001), higher AD (9.24 ± 2.91 vs. 7.77 ± 2.08, *p* < 0.001) than the non-complication group. No statistical difference was found on AW (*p* = 0.576) between the two groups.

**Table 4:** Abdomen anatomic characteristics between complication group and non-complication group after propensity score matching.

	Complication group	Non-complication group	*p* value
Subcutaneous fat thickness (SFT) (mean ± SD) cm	2.72 ± 0.82	2.28 ± 0.89	0.001*
Rectus abdominis thickness (RAT) (mean ± SD) cm	1.27 ± 0.28	1.01 ± 0.24	<0.001*
Abdomen depth (AD) (mean ± SD) cm	9.24 ± 2.91	7.77 ± 2.08	<0.001*
Abdomen width (AW) (mean ± SD) cm	23.84 ± 3.24	24.09 ± 2.26	0.576

*: Significant statistical difference.

### Logistic regression analysis and ROC analysis

**Figure 2:**
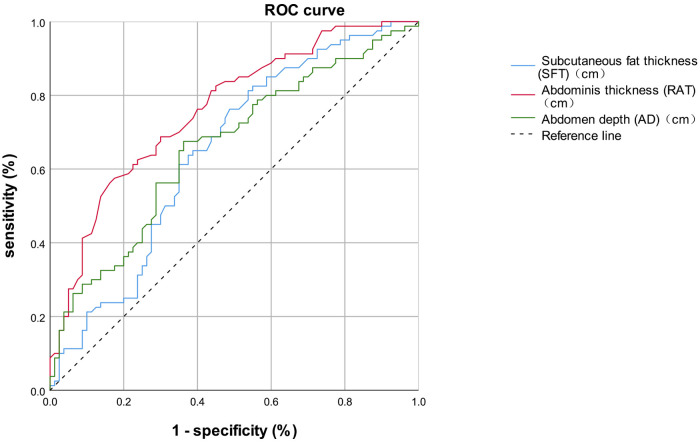
ROC curve.

We subsequently performed Binary logistic regression analysis to identify risk factors for short-term postoperative complications. Elements that were significant in the previous univariate analysis, including preoperative white blood cells, preoperative neutrophil counts, the SFT, the RAT, and the AD, were brought into the logistic regression model. The results shown in Table 5 demonstrated that the SFT (*p* = 0.002), the RAT (*p* < 0.001) and AD (*p* < 0.001) were all the significant risk factors for short-term complications following elective radical resection of colorectal cancer. All these three parameters were introduced into ROC analysis to investigate their predictive ability of short-term postoperative complications, and the results are shown in Table 6 and Figure 2. Compared to the SFT (AUC = 0.648) and AD (AUC = 0.660), the RAT exhibited the best performance in short-term postoperative complication prediction (AUC = 0.761, 95% CI 0.688–0.835, cut-off value = 1.225 cm, sensitivity = 57.5%, specificity = 82.5%, Youden Index = 0.400).

**Table 5:** Abdomen anatomic characteristics between complication group and non-complication group after propensity score matching.

	OR	95% CI	*p*-value
Preoperative WBC	0.824	0.47–1.446	0.033
Preoperative neutrophil counts	1.697	0.868–3.316	0.019
Subcutaneous fat thickness (SFT) cm	1.795	1.097–2.938	0.002*
Rectus abdominis thickness (RAT) cm	57.46	10.485–314.886	<0.001*
Abdomen depth (AD) cm	1.223	1.037–1.443	<0.001*

*: Significant statistical difference.

**Table 6:** Prediction of short-term complication.

	AUC (95% CI)	Cut-off value	Sensitivity	Specificity	Youden index
Subcutaneous fat thickness (SFT) cm	0.648 (0.563–0.734)	2.17	0.763	0.513	0.276
Rectus abdominis thickness (RAT) cm	0.761 (0.688–0.835)	1.225	0.575	0.825	0.4
Abdomen depth (AD) cm	0.660 (0.575–0.744)	8.285	0.675	0.638	0.313

## Discussion

We summarize the main findings of our study here. By comparing 80 patients receiving radical resection of colorectal cancer in the complication group and that in the non-complication group, we discovered that patients with higher preoperative white blood cells, higher neutrophil counts, higher SFT, RA, and AD are at higher risk of short-term postoperative complications. Subsequent logistic regression analysis identified that the SFT, RAT, and AD were independent risk factors for short-term postoperative complications. And further diagnosis power analysis also confirmed that these three parameters could serve as a biomarker for short-term postoperative complications. Our findings suggested that we need to pay attention to the impact of abdomen anatomic characteristics on the incidence of short-term postoperative complications, and remind us to evaluate the risk of short-term postoperative complications in patients with higher SFT, RAT, and AD.

In our study, the short-term postoperative complication includes bleed, infection, anastomotic leakage, ileus, and other surgery-related complications that occurr within 30 days after the operation. As shown in Table 3, postoperative complications can significantly increase the length of hospital stay, which was consistent with the results of previous studies (4). What's more, the incidence of postoperative complications is always associated with an increase of hospital costs and reoperation risk (5, 6). Several studies have shown that postoperative complications in patients with colorectal cancer are associated with poor long-term outcomes (7–9). Therefore, the prediction of the occurrence of postoperative complications is of great clinical significance.

Currently, CT scan is a necessary method for tumour staging and preoperative evaluation before the operation for patients with colorectal cancer. It is easy to figure out the parameters mentioned in our study, including the SFT, RAT, and AD in the CT images. These factors may reflect the underlying state of the patient's abdomen and the nutritional status, which may affect the operation procedure and postoperative recovery. Therefore, we explored the predictive value of these factors for postoperative complications. The measurement of the SFT, RAT, and AD to predict the occurrence of complications can provide a new reference for managing complications without increasing the burden on patients. To rule out the potential co-influence of individual factors on the incidence of postoperative complications in our analysis, we used propensity matching to establish homogeneity of baseline characteristics between the two groups.

Of the three parameters, the RAT is the best predictive factor for postoperative complications of patients with colorectal cancer. Shigemasa Sasaki et al. found that RAT was an independent risk factor for outlet obstruction in males, but there were not enough cases to achieve consistent results in women (10, 11). Tomoaki Kitahara et al. revealed that the risk of outlet obstruction recurrence is high among patients with a thick rectus abdominis muscle (12). Moreover, Song Liu et al. also report that patients with thick rectus abdominis muscle may suffer from a higher risk of surgical site infection. The thicker rectus abdominis muscle theoretically means a richer blood supply, they think that the abundant blood supply of these patients will increase the possibility of bacteria colonization. (13). However, there are also some studies demonstrating that a richer blood supply usually means less risk of infection (14, 15). Therefore, more relevant research should be conducted to clear up this controversial issue. As a possible predictor of postoperative complications, the relationship between rectus abdominis thickness and various complications and the specific reason for the relationship still needs further exploration.

Thicker subcutaneous fat can lead to increased suture tension, which is associated with reduced blood supply and leads to a higher risk of wound liquefaction and delayed wound healing (13). Several previous studies have revealed the association between the high SFT and surgical site infection (16–19). However, in these studies, the cut-off values vary wildly, fluctuating between 10 and 20 mm, which may be related to their use of different methods and standards for measurement. In addition, for the patients with a stoma, thicker subcutaneous fat will make it more difficult to pull out the bowel limbs of the loop ileum without tension and twisting through the narrow subcutaneous cavity. Koichi Tamura et al discovered that SFT is one of the significant predictors of stoma outlet obstruction (20).

Abdomen depth can be used to estimate visceral fat in clinical practice, and some studies have confirmed the relationship between them (21–23). According to some literature reports, visceral fat may be related to a higher risk of postoperative complications for patients with colorectal cancer (24, 25). Alessandro Giani et al. discovered that patients with visceral obesity have a higher risk of anastomotic failure after rectal cancer resection (26). Jun Watanabe revealed that visceral obesity was more highly related to anastomotic leakage and surgical site infection (SSI) than high BMI in patients undergoing surgery for colon cancer (27). In addition, deep abdominal depth may increase the difficulty of surgical exposure and prolong the operation duration, which will increase the risk of postoperative complications.

This study has potential limitations. First, this is a retrospective case-control study in a single centre, and only 160 cases were included in the study after propensity matching. Second, there are some other known predictors associated with short-term postoperative complications which were not evaluated in our analysis. Third, we could not provide a clear explanation of the mechanisms underlying the association between the RAT, SFT, AD, and short-term postoperative complications. However, the results of our study can provide new ideas and possibilities for the prediction and management of short-term postoperative complications for patients with colorectal cancer.

## Conclusion

In conclusion, the risk of short-term postoperative complications following radical resection of colorectal cancer was associated with preoperative RAT, SFT, and AD measured on CT scan. Nevertheless, more multicentre prospective high-quality studies are expected to validate our results in the future.

## Data Availability

The raw data supporting the conclusions of this article will be made available by the authors, without undue reservation.
